# Occult Hepatitis B Virus Infection in Hepatic Diseases and Its Significance for the WHO’s Elimination Plan of Viral Hepatitis

**DOI:** 10.3390/pathogens13080662

**Published:** 2024-08-06

**Authors:** Leticia Bucio-Ortiz, Karina Enriquez-Navarro, Angélica Maldonado-Rodríguez, Jesús Miguel Torres-Flores, Ana María Cevallos, Mauricio Salcedo, Rosalia Lira

**Affiliations:** 1 Medicina y Carcinogénesis Experimental, Universidad Autónoma Metropolitana Iztapalapa, Ciudad de Mexico 09340, Mexico; lebo@xanum.uam.mx (L.B.-O.); karina_enriquez87@hotmail.com (K.E.-N.); 2 Unidad de Investigación Médica en Enfermedades Infecciosas y Parasitarias, UMAE Hospital de Pediatría, CMN Siglo XXI, Instituto Mexicano del Seguro Social (IMSS), Ciudad de Mexico 06720, Mexico; mangimr@yahoo.com.mx; 3 Laboratorio Nacional de Vacunología y Virus Tropicales, Escuela Nacional de Ciencias Biológicas, Instituto Politécnico Nacional, Ciudad de Mexico 11350, Mexico; jtorresf@ipn.mx; 4 Departamento de Biología Molecular y Biotecnología, Instituto de Investigaciones Biomédicas, Universidad Nacional Autónoma de Mexico, Ciudad de Mexico 04510, Mexico; amcevallos@biomedicas.unam.mx; 5 Unidad de Investigación Biomédica Oncológica Genómica, Hospital de Gineco Pediatría 3-A, Instituto Mexicano del Seguro Social, Órgano de Operación Administrativa Desconcentrada (OOAD) Cd Mx Norte, Ciudad de Mexico 07760, Mexico; masava89@gmail.com

**Keywords:** occult hepatitis B virus, liver, hepatic diseases

## Abstract

Liver damage can progress through different stages, resulting in cirrhosis or hepatocellular carcinoma (HCC), conditions that are often associated with viral infections. Globally, 42% and 21% of cirrhosis cases correlate with HBV and HCV, respectively. In the Americas, the prevalence ranges from 1% to 44%. The WHO has the goal to eliminate viral hepatitis, but it is important to consider occult HBV infection (OBI), a clinical condition characterized by the presence of HBV genomes despite negative surface antigen tests. This review aims to provide an overview of recent data on OBI, focusing on its role in the development of hepatic diseases and its significance in the WHO Viral Hepatitis Elimination Plan. Specific HBV gene mutations have been linked to HCC and other liver diseases. Factors related to the interactions between OBI and mutated viral proteins, which induce endoplasmic reticulum stress and oxidative DNA damage, and the potential role of HBV integration sites (such as the TERT promoter) have been identified in HCC/OBI patients. Health initiatives for OBI research in Latin American countries are crucial to achieving the WHO’s goal of eradicating viral hepatitis by 2030, given the difficulty in diagnosing OBI and its unclear association with hepatic diseases.

## 1. The Liver and Pathologies

The liver, the body’s largest organ, carries out over 500 functions, which include metabolism and detoxification. Exposure to different factors can impair hepatocyte function, resulting in conditions ranging from mild to severe, such as liver failure, cirrhosis, and hepatocellular carcinoma (HCC) [1].

Several liver pathologies such as non-alcoholic fatty liver disease (NAFLD), alcoholic liver disease (ALD) [2] and non-alcoholic steatohepatitis (NASH) have been linked to cirrhosis and HCC progression [3]. While the exact mechanism of triglyceride accumulation remains unclear, lipid overflow has been proposed as an initial trigger. This lipotoxicity, along with other factors (second hit), initiate hepatic inflammation and tissue fibrosis, potentially leading to NASH and subsequently to cirrhosis [4].

**Figure 1:**
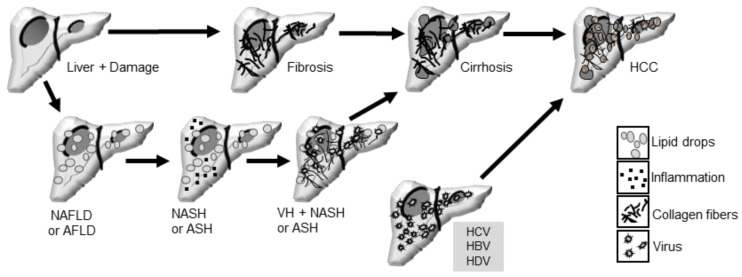
Liver pathologies and their progression to more severe states include NAFLD (Non-Alcoholic Fatty Liver Disease) and AFLD (Alcoholic Fatty Liver Disease), which can progress to steatohepatitis and induce fibrosis. NASH (Non-Alcoholic Steatohepatitis) and ASH (Alcoholic Steatohepatitis) can further progress to cirrhosis, potentially leading to hepatocellular carcinoma (HCC). Additionally, viral hepatitis (VH), including infections with HCV (Hepatitis C Virus), HBV (Hepatitis B Virus), or HDV (Hepatitis D Virus), can favor progression to cirrhosis and HCC.

Hepatitis, defined as liver inflammation, can manifest as acute or chronic, and may be caused by toxic chemical substances (alcohol or drugs) or infection with hepatitis viruses: hepatitis A virus (HAV), B (HBV), C (HCV), D (HDV), and E (HEV) [5]. Chronic infections with HBV and HCV are of particular interest because they have been associated with an increased risks of liver disease progression to cirrhosis and liver cancer, making them an important public health problem [5] (Figure 1). 

Liver damage can progress to cirrhosis and liver cancer. Cirrhosis, characterized by extensive collagen deposition in the extracellular matrix of hepatocytes, comprises liver function and can lead to mild or advanced fibrosis, which can cause HCC [6]. Liver cancer ranks fourth among causes of death globally, occurring more frequently in countries with low human development indexes (Africa, Asia) [7] (Figure 1). Primary liver cancer includes hepatocellular carcinoma (HCC) in 75–85% of cases [8], intrahepatic cholangiocarcinoma (10–15%), and other rare cancers [7]. Chronic HBV infection (HBC) is the main cause of HCC [8] worldwide, although other factors such as aflatoxin-contaminated food, a fat-rich diet (obesity in the West), smoking, type 2 diabetes [7], and metabolic syndrome [8] also contribute to its development. HCCs are complex ecosystems incorporating non-tumor cells such as those of the immune system. Thirty percent of early-stage HCCs show genomic evidence of immune activation, while 25% show no immune infiltration. HCCs are often highly heterogeneous, with three growth patterns reported: trabecular, pseudoglandular (or pseudoacinar), and solid, which can be found in the same tumor area [8].

## 2. Hepatitis B Virus Characteristics

HBV is an enveloped DNA virus from the *Hepadnaviridae* family [9]. The genome is a relaxed circular DNA (rcDNA) molecule, partially double-stranded, ranging from 3.1 to 3.3 Kb. The positive strand is incomplete, while the negative strand is complete, containing two identical direct repeat sequences (DR1 and DR2) [10]. It encodes four overlapping open reading frames (ORFs): the core ORF (C), which consists of two regions, precore and core (preC/C), that code for the e antigen (HBeAg) and the core protein (HBcAg); the surface protein ORF (S), which is structurally and functionally divided into three regions (PreS1, PreS2, and S), encoding for the large (LHBs), medium (MHBs), and small (SHBs) surface proteins, the latter of which is also known as the surface antigen (HBsAg); the polymerase ORF (P), which encodes for the polymerase (Pol); and ORF X, which encodes the X protein (HBx). The rcDNA converts into covalently closed circular DNA (cccDNA), which is transcribed into four viral RNAs from different HBV promoters. The 3.5 Kb RNA translates into C and P; the 2.4 Kb RNA into LHBs; the 2.1 Kb RNA into MHBs and SHBs; and the 0.7 Kb RNA translates into HBx [10,11]. The viral polymerase, which has reverse transcriptase (RT) activity, participates in the replication of the partially double-stranded DNA genome.

HBV polymerase lacks 3′−5′ exonuclease activity, making it error-prone and leading to genetic variability. Ten genotypes (A–J) and nearly 40 subgenotypes have been identified. Each genotype has more than an 8% genetic divergence across the entire genome and may differ in terms of geographical distribution, transmission route, and virological characteristics [12].

Genotype C has been associated with a higher risk of developing severe liver disease, cirrhosis, and HCC compared to genotype B [13,14,15]. However, genotype B has been linked to the development of HCC in children with chronic HBV infection [16]. In genotypes C and D, a high frequency of mutations in the basal core promoter (BCP), lower response to interferon therapy, faster progression to liver fibrosis, and HCC have been reported [12]. Chronic HBV and HCV infections account for 60–70% of HCC cases [17], while cirrhosis cases are 42% and 21% for HBV and HCV, respectively. In the Americas, the overall prevalence (HBV + HCV) ranges between 1% and 44% [18].

## 3. Global Strategy to Eradicate Viral Hepatitis, Focusing on the Context of OBI

At the 63rd World Health Assembly in 2010, the WHO recognized the significant global burden of disease and mortality caused by viral hepatitis. In response, in 2016, the WHO set the ambitious goal to eliminate viral hepatitis worldwide by 2030. To achieve this, various initiatives have been implemented, focusing on improving prevention through early diagnosis, effective treatment, and comprehensive, multidisciplinary management of hepatitis patients. A delayed diagnosis can result in the progression to severe diseases, increased mortality, decreased quality of life, and higher healthcare costs. Interrupting transmission is crucial for eliminating viral hepatitis. Additionally, awareness and education campaigns about viral hepatitis have been conducted to increase public understanding [19,20].

### 3.1. Occult Hepatitis B Virus Infection

Although the primary focus is on eliminating HCV and HBV, the entity of occult hepatitis B infection (OBI) should not be overlooked. OBI is defined by the presence of replication-competent HBV-DNA in the liver of individuals negative for HBsAg and with low viral load (<200 IU/mL). Diagnosing OBI presents challenges due to the intermittent detection of HBV-DNA in blood and the lack of standardized and validated sensitive molecular assays for VL [21,22]. The diagnostic methods used include the detection of HBV-DNA in blood by nested PCR techniques, real-time PCR assays, or digital PCR assays [23]. Ideally, detection would involve identifying replication-competent HBV-DNA in the liver through molecular techniques, although no validated tests currently exist for this purpose, posing an obstacle to the accurate diagnosis of OBI.

The epidemiology of occult hepatitis B infection (OBI) varies, with prevalence rates associated with differing endemicity statuses across studies due to the use of different methods and sensitivities in determining HBV-DNA viral load (VL). The prevalence of OBI ranges from 1 to 87% in different regions of the world. A recent meta-analysis concluded that the prevalence of OBI among blood donors is correlated with the endemicity of the disease, being 0.06%, 0.12%, and 0.98% in low-, intermediate-, and high-endemicity countries, respectively [24]. In high-risk groups, such as those with HCV or HIV co-infection, HCC, and cryptogenic cirrhosis, the prevalence was 5.5%, 5.2%, and 12% in countries with low, intermediate, and high endemicity, respectively [24]. In the Americas, few studies have reported an approximate prevalence of 16% [24]. In Mexico, a prevalence between 18.7 and 49% has been reported in HIV co-infected patients [25,26]. Recently, only one study from Brazil has been searching for the presence of OBI [27]. Although an association between OBI and HCC has been reported in case–control and prospective cohort studies [28,29], there is no registry of the prevalence of OBI in patients with HCC. 

The lack of validated tests for diagnosis and the limited information on prevalence in underdeveloped countries are significant challenges in achieving the WHO’s goals for eradicating viral hepatitis. Given the multifactorial nature of OBI, various mechanisms have been proposed to be involved in the persistence of the virus, including co-infections with HCV and HIV, factors related to immune response, genome integration, and mutations in the HBV genome [30,31]. Additionally, host epigenetic modifications and immune control of gene expression have been implicated as important factors for cccDNA persistence due to the suppression of viral replication in OBI [32]. However, this review did not address this aspect.

### 3.2. OBI and Hepatic Diseases

Additionally, intrinsic host factors, such as obesity, pose a risk for triggering progressive liver damage, from non-alcoholic fatty liver disease (NAFLD) to non-alcoholic steatohepatitis (NASH), cirrhosis, and HCC. OBI is a potential cofactor to accelerate the progression of liver disease to cirrhosis and HCC due to different etiological causes [28,33], and various studies in animal models and clinical reports suggest that the same direct and indirect mechanisms described for HBV are involved in the transformation to HCC in OBI [28,34]. A study in obese patients undergoing bariatric surgery showed a prevalence of 12.8% of OBI, and they conclude that an obese individual with OBI has a higher risk of developing NASH, likely accelerating hepatic inflammation processes [35]. Another study evaluated the impact of alcohol intake and OBI infection on the severity of NAFLD, reporting that the presence of anti-core is a factor associated with advanced fibrosis and suggesting that OBI could negatively affect the development of NAFLD. This suggests the potential impact of OBI in NAFLD, which should be considered as a factor for progressive liver damage [36].

The association between HBV and HCC is well established [37], but in Mexico and in Latin American countries with low HBV prevalence, few studies have been conducted, resulting in a low incidence of patients with OBI and HCC [24,38]. Specific genetic and environmental factors of the Mexican population may influence this difference [39]. Therefore, there is still a need for novel research and a comparative analysis of multiple risk factors interacting among different populations [40].

### 3.3. Mutations in HBV Genome Associated with Liver Diseases

Among the various pathogenic mechanisms of OBI for developing liver diseases, particularly in HCC, it has been suggested that the level of viral replication, genotype, genetic variants, and mutations in the genome influence the outcome [31,41,42]. In this review, we compile various reports describing mutations in the HBV genome in cases of OBI and chronic HBV infection (HBsAg+) associated with the development of liver diseases (Table 1). HBV has been associated with variants in the PreS/S regions that can cause liver disease through various mechanisms [43,44]. Deletions in the PreS1 and PreS2 regions result in an unbalanced production of mutant envelope proteins, inducing accumulation in the endoplasmic reticulum (ER), generating reactive oxygen species (ROS), DNA damage, genomic instability, and activating signaling pathways related to hepatocarcinogenesis [45,46,47].

The F141L mutation in the PreS2 region increases the risk of HCC in patients with HBV/C infection, and other mutations in PreS2 directly activate signaling pathways associated with tumor development [76]. PreS mutants have also been shown to induce dysplasia in hepatocytes and lead to the development of HCC in animal models [46].

The G185R and S210N mutations in the S gene, M1I and Q2K in PreS2, and G1721A in the Enhancer II (EnhII) region were more common in Taiwanese patients with HCC and OBI than in those with HCC and HBC infection [77]. The pattern of two PreS2 mutations and one EnhII mutation (G1721A, M1I, and Q2K) has been proposed as a viral marker of HCC in OBI carriers. Khan et al. reported other mutations in the EnhII/BCP regions, both individual and combined, associated with the development of HCC [69]. Other natural mutations identified in the BCP and PreC regions are associated with the development of HCC [78]. These mutations could be proposed as potential biomarkers to predict the onset of HCC and facilitate timely diagnosis and treatment.

## 4. The HBx Protein, ER Oxidative Stress, and HBV-DNA Integration

The X protein (HBx) modulates various cellular processes and signaling pathways involved in the development of HCC by inactivating tumor suppressor regulators, such as p53, and regulating metalloproteinases related to metastasis [79,80,81]. Specific mutations in the X gene of HBV, such as the X8Del, an 8-bp deletion in the C-terminal region, have been associated with OBI by reducing the secretion of HBsAg and virions [41]. In addition to the potential transforming capability of HBx, chromosomal instability resulting from the random integration of HBV-DNA into the chromosome has been proposed [82,83,84]. Furthermore, it has been suggested that low levels of replication of the occult virus can also induce constant necroinflammation of the liver resulting in cirrhosis progression [85].

In hepatocarcinogenesis derived from a cirrhotic liver, hepatocyte chromosomes present shorter telomeres and the p53 mutation, which have been associated with the development of early liver neoplasms [86,87,88]. The activation of stellate cells during cirrhosis induces the production of cytokines, growth factors, and oxidative stress, affecting hepatocyte proliferation and possibly contributing to tumor formation. The key oncogenic pathways involved include PI3K/Akt, myc, Wnt/β-catenin, c-Met, and hedgehog [86].

### 4.1. Endoplasmic Reticulum Stress and HBV Mutations

The high demand for viral protein synthesis in infected hepatocytes induces endoplasmic reticulum (ER) stress, a risk for maintaining their homeostasis. The unfolded protein response (UPR) is activated by the induction of protein kinase RNA-like endoplasmic reticulum kinase (PERK), IRE1, and ATF6. Under stress conditions, PERK phosphorylates eIF2a, inducing the expression of apoptosis-related genes. GRP78, bound to PERK, dissociates during stress, inducing liver damage. IRE1 kinases activate ASK−1, JNK, and p38 MAPK, inducing apoptosis. Deletions in PreS in the hepatitis B virus (HBV) generate altered envelope proteins that accumulate in the ER, inducing ER stress and abnormal secretion of HBsAg [89].

Although PreS deletions have been considered an independent risk factor for HCC, PreS1 mutants have shown increases in GRP78 and GRP94 expression in in vitro studies, while PreS2 mutants decrease their expression [90]. The observation of hepatocyte cytoplasm infected with PreS mutants as ground glass highlights significant alterations in cellular morphology [90].

Recently, it has been reported that HBxAg also induces ER stress, and that in genotype C, high levels of reactive oxygen species (ROS) and ER stress are expressed (compared to genotype B), as well as high levels of GRP78, promoting liver damage associated with HCC [89]. Elevated ROS levels can cause mutations, promote proliferation, evade apoptosis, angiogenesis, metastasis, and interfere with the pathogenesis and progression of HCC by causing damage to DNA, RNA, lipids, and proteins [91,92,93]. In mammals, ROS can promote tumor development and progression through two pathways: the mitogen-activated protein kinase (MAPK) and the phosphatidyl-inositol 3-kinase/protein kinase-B/target of rapamycin (PI3K/AKT/mTOR) [91]. The MAPK pathway is activated by cytokines through a phosphorylation of MAP3K and p38 mitogen-activated kinase proteins or c-Jun N-terminal kinases (JNK). A decreased expression of p38 and dual MAP kinase 6 (MKK6) was associated with larger HCC lesions [94]. Interestingly, phosphorylated kinases pJNK1 and pJNK2 have been detected in most HCC patient samples. The activation of MEK/ERK is also related to the proliferation of HCC cells and the abnormal activation of Ras/Raf/MEK/ERK in HCC patient samples [94].

ROS can act on cell proliferation by increasing the phosphorylation of PI3K or AKT or by decreasing the PTEN levels. In HCC biopsies, the PIK/AKT/mTOR pathway was found to be over-regulated, with a loss of PTEN and activation of AKT, showing an association with low differentiation, high proliferation, and intrahepatic metastasis. Although these ROS-activated pathways are highly efficient, cancer cells have two response mechanisms to reduce ROS overproduction and its toxic effects: the Kelch-like ECH-associated protein 1 (KEAP1)-nuclear factor erythroid 2-related factor 2 (Nrf2) pathway [93], and the GSH metabolism capable of reducing oxidative stress via aldehyde dehydrogenase (ALDH) [94].

In Huh7 cells transfected with PreS mutants (LHBs), ROS levels increase, potentially causing genomic instability and leading to HCC. PreS mutants also upregulate COX−2 and cyclin A, which are associated with cell cycle progression induction in several types of cancer; elevated COX2 mRNA levels were observed in HCC biopsies. Additionally, it has been reported that the nuclear transcription factor (NF-kB) is required to activate the COX2 promoter, particularly the p65 subunit that translocates to the nucleus during ER stress, and the inhibition of NF-B abrogates COX2 induction, suggesting that PreS mutants can induce COX−2 via NF-B and p38MAPK [89].

Oxidative stress and the high energy demand for tumor proliferation create a microenvironment that modulates the immune response. NK cells, which participate in the innate response against viral infections, produce cytokines that eliminate infected and tumor cells. During HBV infection, the cytokine balance is disrupted, suppressing the immune response and affecting NK cells. TNF-α and IL−6, secreted by macrophages, also influence tissue regeneration and HCC. The lack of IL−6 increases HCC and reduces NK cells, suggesting their interaction, although the mechanism is unknown [95].

### 4.2. Viral DNA Integration and HCC

The integration of viral DNA into the host genome is one of the primary mechanisms in the development of liver cancer, particularly associated with HBV infection [96,97]. It has been observed that hepatocytes can harbor multiple copies of genome integrated HBV-DNA, even after the elimination of the cccDNA HBV through antiviral or immunological therapies [98]. Integration, which occurs early in HBV infection and persists during chronic infection, primarily occurs in regulatory regions of the genome, inducing genomic instability and altering the expression of cancer-related genes [99]. It has been reported that HBV integration does not generate transcripts competent for viral replication, however, it is a stable source of viral RNA and proteins that can contribute to viral persistence [98]. In patients with HCC, viral DNA integration has been observed near hepatic oncogenes and in coding regions of the human genome [100]. Additionally, integration has been identified in the TERT promoter and regions enriched with long interspersed nuclear elements (LINEs) and satellite regions [101]. These events can activate signaling pathways such as Wnt/β-catenin, reduce miR−122 levels, and promote HCC development [102].

In patients with OBI, viral integration is associated with accelerated hepatocarcinogenesis, although its impact on those who do not develop HCC is still unclear [22,103]. The presence of cccDNA in the liver of OBI patients can lead to low but constant viral transcription and replication [96], which could also influence the lack of detection of HBsAg [104,105]. Although the transcriptional control mechanisms of cccDNA are not yet clear, they could play a role in the persistence of the virus in this clinical context. 

The persistence of viral DNA in OBI patients can contribute to liver inflammation and fibrosis, especially in combination with other risk factors such as HCV infection or alcohol consumption [106]. In a study on patients with cryptogenic HCC, 73% had OBI, and HBV-DNA was detected more frequently in tissues adjacent to the tumor [84]. In another study with 90 patients, 69% had OBI, but only half had cccDNA in the liver, while in nearly 90% of patients with undetectable cccDNA, integrated viral DNA was identified [107]. 

## 5. Immune Response and Biomarkers

Immune control is crucial in patients with OBI, as evidenced by the reactivation of the virus in those receiving immunosuppressive therapy with anti-CD20 monoclonal antibodies or hematopoietic stem cell transplantation [108]. Immune responses in OBI are constantly stimulated by low but persistent concentrations of viral antigens, which can lead to different T cell response profiles [109]. It has been suggested that the persistence of transcriptionally mutated cccDNA is regulated by epigenetic mechanisms [32].

Although it is not clear whether OBI accelerates progression to cirrhosis and HCC in patients with pre-existing liver disease due to other factors such as HCV infection, alcohol, or steatohepatitis [38], it has been suggested that OBI may result from blood transmission [110], organ transplantation, or reactivation during immunosuppressive treatment and after the discontinuation of immunosuppressive therapy in HIV-infected patients [108] or those receiving chemotherapy [111]. On the other hand, the evidence to determine if OBI could accelerate the progression to cirrhosis and HCC in patients with previous liver disease, caused by other factors such as HCV infection, alcohol, or steatohepatitis, remains insufficient [38]. The treatment of HCC varies according to the stage of the disease and may include surgical procedures [8] or chemotherapy [112], although drug resistance remains a significant challenge [94]. Early detection of HCC through biomarkers, as well as the evaluation of viral infections, can improve the ability to identify OBI and contribute to the eradication of viral diseases.

Although the investigation of biomarkers in HCC tumor tissues is an alternative that could offer more treatment options, the sensitivity and specificity of these biomarkers are still in the process of validation and application [86,112]. However, this strategy, together with tests to detect viral infections, may be the key to improving outcomes in the eradication of viral diseases in the region. Finally, it is essential to conduct more research to better understand the association between OBI and liver damage, especially in the context of the World Health Organization’s plan to eradicate viral hepatitis by 2030 in Latin American countries where few studies have been conducted, such as Mexico, Argentina, Brazil, Venezuela, and Colombia.

## 6. Conclusions

The liver can experience damage that leads to various pathologies, eventually resulting in cirrhosis and HCC. This damage is strongly related to viral infections, among which OBI is expected to have a higher incidence in the future. This review has considered HBV genome mutations as a relevant factor in the development of HCC, including some already described in OBI, such as mutations in the ORF-S and ORF-C as well as deletions in PreS/S, and in the ORF-X, which induce ER stress, an increase in oxidative stress, and consequently the activation of signaling pathways associated with HCC.

Certainly, HBV vaccination programs have been successful worldwide, leading to a significant reduction in the prevalence of chronic HBV. However, in Latin American countries, the vaccination efforts have not achieved the anticipated impact. Therefore, studies focusing on the detection of anti-HBc antibodies are crucial. These studies will help identify the true prevalence of HBV in the population, which is essential for developing effective strategies to improve vaccination coverage and outcomes. Additionally, OBI is an entity that is not well known but should be considered for the WHO Viral Infection Eradication Plan, established for Latin American countries by 2030.

In summary, the elimination of viral hepatitis by 2030 is an ambitious goal, but the challenges in the precise diagnosis of OBI, the lack of data in underdeveloped countries, and the complexity of associated factors require coordinated global efforts. Continuous research and understanding of the underlying mechanisms are essential to progress towards the eradication of diseases caused by the hepatitis B virus.

## Figures and Tables

**Table 1:** Mutations in the Pre-S/S, PreC/C, and X reading frames associated with liver diseases.

Gen/Region	Genotype	Mutation	Function	OBI/HBsAg+	Pathology	Reference
**ORF** **-** **S**						
PreS1	A	Ins of CAACA at nt 503, T Del at nt 523 and 1368–1525	Alteration of regulatory regions	OBI	HCC	[48]
	B, C	Dels in aa (31–80, 67–55, 92–119, 102–119, 1–83)	Immune escape	OBI	HCC in children	[49]
	D, A	T40S, P124K, L54D, G76A, Dels in aa 4,8,17 and 86	Descriptive study	OBI	HCC/Cirrhosis	[50]
	C	W4R, L30S, I84T, Q118R/Stop	Evasion of the immune response	OBI	HCC	[51]
PreS2	B, C	Dels in aa (1–5,7–20,5–21,12–29,1–12,8–23,1–21)	Evasion of the immune response (B-cell epitope)	OBI	HCC in children	[49]
	D, A	P41H, P66L			HCC	[50]
	C	N5D, S5F/D			HCC	[51]
	C	Deletions, M1I	Alteration of regulatory regions. Immune escape	HBsAg+	HCC	[52]
S	C	W36L, T47K, N52D, V184A, F220L	Decrease HBsAg secretion, ER stress, and ROS	OBI	HCC	[53]
	C	W74L, L77R	Decrease HBsAg secretion	OBI	HCC	[54]
	C	L42F/S, Y200F/S	Descriptive study. Evasion of the immune response	OBI	HCC	[51]
	A, D	F19L, S59F, P24L and Q129H	Descriptive study	OBI	HCC	[50]
	A, D	P203, S210R	Decrease HBsAg secretion, increase cell proliferation	HBsAg+	HCC	[55]
CTL epitope	A, D	N40S, S45A, V47A	Immune escape, vaccine evasion, disease progression	OBI	HCC	[48]
“*a*” det (124–147)	A, D	T125M	Immune escape	OBI	Cirrhosis	[48]
MHR	B, C	Q101K, T115A, K122N, T123A, T126N, Q129N, G130R, T131I, M133T, F134L, C138Y, K141E, P142S, G145R, N146S, C147F/R	Naturally occurring immune escape variants	OBI	Cirrhosis, blood donors	[56]
Outside **MHR (N-t and C-t regions)**		41, 44, 48, 93, 96, 97, 171, 175, 176, 178, 185, 190, 207, and 213 positions	Immune escape variants	OBI		[57,58]
**ORF-C**						
PreC	A, D	A1814T/C, T1815C, G1816T/A (M1L/T/I)	Reduce the expression of HBeAg	HBsAg+	HCC	[59,60]
	A	G1862T (V17F)	Reduce HBeAg expression	HBsAg+	HIV	[60]
	A, C, D	G1896A (W28stop)	Inhibits the production of HBeAg	HBsAg+	HCC	[61,62,63,64]
Core	B, C	C1913A, C1914A/T, G1915A/C (P5T/L/H)	Induces ERO	HBsAg+	HCC	[53,61,63]
	C	E83D	Immune escape		HCC	[61]
		I97F	HBcAg assembly		HCC	
Hot spot region	A, C, D	C2198A (L100I)	Immune evasion by CD4 T cells, disease progression	HBsAg+	HCC	
(aa 81–105)		W62R, P50H, S74G	Decreases secretion of HBcAg and HBeAg		Blood donor	[65]
**ORF-X**						
EnhII/BCP	B, C	T1753A/C/G (I127T/N/S)	HCC predictor	HBsAg+	HCC	[66,67,68]
	B, C, D	G1762T (K130M)	It partially contributes to cellular stress, tissue inflammation	HBsAg+	HCC, fibrosis, cirrhosis	[68,69,70]
	C	A1762T	Decreases life expectancy	HBsAg+	HCC	[71,72]
	B, C, D	G1764A (V131I)	Cellular stress, tissue inflammation	HBsAg+	HCC, fibrosis, cirrhosis	[68,69,70,73]
	C	Double mutation A1762T/G1764A	Cellular stress, tissue inflammation, decreases life expectancy	HBsAg+	HCC	[72,74,75]
	D	Triple mutationA1757/T1764/G1766	Associated with the development of HCC			[69]
	D	Quadruple mutationT1673/G1679/T1773/G1775	Synergistic effect on HCC development			
**C-t region**	C	X8Del	Reduces HBsAg and virions secretion	OBI	None	[41]

Dels, deletions; Ins, insertion; “*a*” det, “*a*” determinant; HCC, hepatocarcinoma; Dels; OBI, occult hepatitis B virus infection; N-t, amino terminal region; C-t, carboxy terminal region.

## Data Availability

No new data were created or analyzed in this study. Data sharing is not applicable to this article.
